# Probiotic intervention modulates volatile sulfur compound-associated pathways and oral microbiome balance in ligature-induced periodontitis rats

**DOI:** 10.1080/20002297.2026.2705080

**Published:** 2026-08-20

**Authors:** Md Ariful Haque, Hye Kim, Jin Seock Moon, Seong Rok Sim, Md Abdur Razzak, Sehyeon Song, Jong Ik Jeon, Myeong Soo Park, Seockmo Ku

**Affiliations:** a Department of Food Science and Technology, Texas A&M University, College Station, TX, USA; b Research Center, BIFIDO Co., Ltd, Hongcheon, South Korea

**Keywords:** Halitosis, probiotics, periodontitis, ligature-induced, oral dysbiosis

## Abstract

**Background:**

Halitosis is driven by volatile sulfur compounds (VSCs) produced by anaerobic oral bacteria and commonly worsens with periodontitis. We investigated whether the probiotic complex Complex OK suppresses VSC pathways, modulates oral dysbiosis, and reduces inflammation.

**Materials and methods:**

Complex OK was evaluated *in vitro* for downregulating the VSC-related *mgl* gene and antagonism against malodor pathogens using a 1:1 co-culture of *Lactobacillus gasseri* HHuMIN D and *Lacticaseibacillus paracasei* OK. *In vivo*, ligature-induced periodontitis was created in 45 male Sprague-Dawley rats assigned to healthy control, periodontitis control, or low-, medium-, and high-dose groups (0.2, 2, and 20 mg/head/day, corresponding to approximately 10^7, 10^8, and 10^9 CFU/head/day, respectively) treated for 5 weeks. Endpoints included body weight, saliva secretion, tongue coating index (TCI), alveolar bone loss, systemic IL-1β, and oral microbial composition.

**Results:**

Complex OK reduced *mgl* expression and inhibited *Streptococcus mutans* (98.2%), *Fusobacterium nucleatum* (91.2%), *Porphyromonas gingivalis* (99.1%), and *Prevotella intermedia* (96.6%). High-dose supplementation increased body weight (329.02 ± 14.59 g) and saliva secretion (4.03 ± 2.06 mg), lowered TCI (6.40 ± 2.60), decreased *Fusobacterium* abundance, reduced IL-1β, and showed a non-significant trend toward reduced alveolar bone loss compared with periodontitis controls.

**Conclusion(s):**

Complex OK may represent a noninvasive probiotic strategy for modulating halitosis-associated molecular pathways, supporting oral microbiome balance, and reducing periodontal inflammation, with potential as an adjunctive therapy for periodontal care pending further clinical validation.

## Article highlights

- Complex OK suppresses *mgl* genes, reducing sulfur compound levels linked to halitosis.
- Probiotics block oral pathogens' growth and stop them from binding to epithelial cells.
- Tongue coating index declined, indicating less biofilm and better oral cleanliness.
- Probiotics modulates microbiota, reducing harmful taxa such as *Fusobacterium*, *Veillonella*.
- Probiotics lowered IL-1β, showing systemic anti-inflammatory effects on the body.

## Graphical abstract

**Figure:**
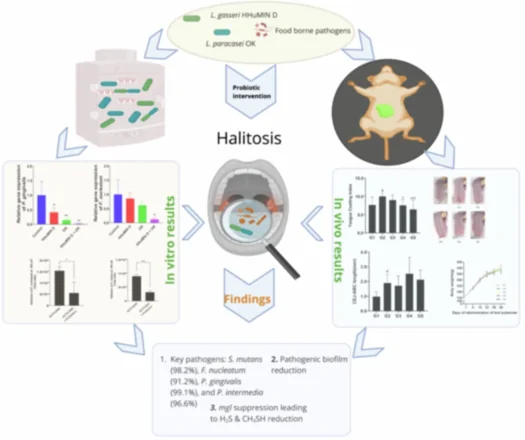

## Introduction

Halitosis, or bad breath, affects approximately 30–50% of the global population and has substantial social and psychological consequences [1,2]. It is primarily associated with volatile sulphur compounds (VSCs), including hydrogen sulphide (H₂S) and methyl mercaptan (CH₃SH), which are generated through the anaerobic metabolism of sulphur-containing amino acids by oral bacteria. These compounds are particularly abundant in periodontal disease, where pathogens such as *Fusobacterium nucleatum*, *Porphyromonas gingivalis*, *Veillonella*, and *Streptococcus mutans* contribute to biofilm formation, periodontal inflammation, and VSC production [3,4].

Conventional approaches, including mechanical debridement and antimicrobial agents such as chlorhexidine and cetylpyridinium chloride, can reduce bacterial burden but often fail to restore oral microbial homoeostasis and may disrupt the resident microbiota [5]. Consequently, probiotics have emerged as a promising microbiome-based strategy for improving oral health [6]. Defined as ‘live microorganisms that, when administered in adequate amounts, confer a health benefit on the host’ [7,8], probiotic lactic acid bacteria (LAB) have been shown to inhibit oral pathogens through competitive adhesion, bacteriocin production, and immune modulation while suppressing pathways involved in VSC production [9]. For example, *Weissella cibaria* CMU and *Lactobacillus reuteri* have been reported to downregulate the *mgl* gene in *P. gingivalis*, thereby reducing methyl mercaptan production, a major contributor to oral malodor [10,11].

Among candidate probiotics, *Lactobacillus gasseri* HHuMIN D and *Lacticaseibacillus paracasei* OK have demonstrated antimicrobial activity against oral pathogens and the ability to suppress the expression of the methionine γ-lyase (*mgl*) gene, a key enzyme involved in VSC biosynthesis [4]. These strains have also been shown to inhibit bacterial adhesion and biofilm formation, two critical processes in the development of periodontal disease [12,13]. In vivo studies using ligature-induced periodontitis models have further demonstrated improvements in tongue coating index (TCI), microbial diversity, and inflammatory cytokine profiles following probiotic supplementation [14]. The high-dose group demonstrated a clear reduction in pathogenic genera including *Veillonella* and *Escherichia-Shigella*, correlating with improved oral health markers and reduced VSC-related symptoms.

Given the growing concerns over antibiotic resistance and the limitations of antiseptic rinses, probiotic interventions offer an eco-friendly, sustainable, and microbiome-preserving solution for oral health management [15]. By modulating both microbial ecology and immune responses, probiotics like *L. gasseri* HHuMIN D and *L. paracasei* OK may serve as functional alternatives to conventional antimicrobial strategies.

Although probiotics are primarily recognised for their direct interactions with the oral microbiota, accumulating evidence suggests that orally administered probiotics may also influence oral health through systemic mechanisms involving oral–gut microbiome crosstalk and immune modulation. Previous studies have proposed that probiotic-mediated regulation of inflammatory cytokines, enhancement of mucosal barrier function, and modulation of host immune responses may indirectly affect the oral microbial ecosystem and periodontal inflammation. Although these mechanisms provide a biologically plausible rationale for evaluating probiotic administration via oral gavage, they have not been directly established in the present study and are presented here as hypotheses supported by the existing literature.

Given the increasing prevalence of antimicrobial resistance and the limitations of conventional therapies for halitosis and periodontal disease, targeted probiotic interventions have emerged as a promising microbiome-based strategy for promoting oral health. Therefore, the present study aimed to evaluate the probiotic potential of *Lactobacillus gasseri* HHuMIN D and *Lacticaseibacillus paracasei* OK in a ligature-induced periodontitis model by assessing surrogate biomarkers associated with halitosis and periodontal health, including mgl gene expression, inhibition of pathogen growth and adhesion, tongue coating index, inflammatory cytokines, and oral microbiome composition. Through these assessments, this study sought to provide insight into the potential biological effects of this probiotic combination while laying the foundation for future mechanistic investigations.

## Material & methods

### In vitro studies of halitosis

#### Bacterial strains and growth conditions

*Lactobacillus gasseri* HHuMIN D and *Lacticaseibacillus paracasei* OK were anaerobically cultured in MRS broth (BD Difco™) supplemented with 0.05% L-cysteine at 37 °C. *S. mutans* KCTC 3065 was cultured in BHI broth (BD Difco™), while *F. nucleatum*KCTC 2640 was cultured in RCM broth (BD Difco™) at 37 °C under anaerobic conditions. *P. gingivalis* KCTC 5352 and *Prevotella intermedia* KCTC 15693 were anaerobically cultured at 37 °C in KCOM1 medium, which consisted of tryptic soy broth (7.5 g), yeast extract (5 g), 0.025% resazurin, 0.05% hydrogen chloride, 5 μg/mL hemin, and 2 μg/mL vitamin K1.

#### Measurement of *mgl* gene expression

*L. gasseri* HHuMIN D or *L. paracasei* OK was cultured in MRS broth (0.05% L-cysteine), and the obtained culture supernatant (20%) was incorporated into KCOM1 medium containing 1 × 10^7^ CFU of *P. gingivalis*, followed by anaerobic incubation at 37 °C for 18 hours. A mixed supernatant was prepared by combining the culture supernatants of *L. gasseri* HHuMIN D and *L. paracasei* OK in a 1:1 ratio. For the combined supernatant treatment, 20% of the mixed supernatant was added to KCOM1 medium containing *P. gingivalis*, and the mixture was anaerobically incubated at 37 °C for 18 hours. The control group replaced 20% of the culture supernatant with a 20% diluted MRS broth (0.05% L-cysteine) under identical conditions. After incubation, bacterial cells were collected from 1 mL of culture, and genomic DNA was extracted for quantification by real-time PCR. The primers used for bacterial quantification (Table 1) [10,12] included specific primers targeting the *mgl* gene of *P. gingivalis*: TCGTGCTTATGAGCGATGTC and GGAAGTCACCCTCGTGGATA. The obtained Ct values were analysed using the 2-ΔΔCt method to determine the relative fold change in *mgl* gene expression compared to the control.

**Table 1.** The primers used for bacterial quantification.

Bacterial strain	Primers (5’-3’)	Length (bp)	Reference	
*L. gasseri* HHuMIN D	GCATTAATCCTAGCAGCACGC	21	This study	
	TAGGCATTTGAGCACCTCCTT	21		
*L. paracasei* OK	GCTGCAAAAAGCCATCGACA	20	This study	
	CGATTGACCGTGATTTCGGC	20		
*S. mutans*	CTACACTTTCGGGTGGCTTG	20	[12]	
	GAAGCTTTTCACCATTAGAAGCTG	24		
*F. nucleatum*	AGAGTTTGATCCTGGCTCAG	20	[12]	
	GTCATCGTGCACACAGAATTGCTG	24		
*P. gingivalis*	TGTAGATGACTGATGGTGAAAACC	24	[10]	
	TTTAGAGATTCGCATCCGGT	20		
*P. intermedia*	TGGTTCGATAACGGCAGCAT	20	This study	
	ACGTAGCCAACAGCAGGAAA	20		

For *F. nucleatum*, a similar method was applied using KCOM1 medium for anaerobic culture. The *mgl* gene of *F. nucleatum* was amplified using the following primers: CGTATGGAAAGACACTGTGCA and TTACCTGCTTCAAAGCCACC. The Ct values were analysed using the 2-ΔΔCt method to determine the relative fold change in *mgl* gene expression compared to the control.

#### Antimicrobial activity

The antimicrobial activity of *L. gasseri* HHuMIN D and *L. paracasei* OK against oral pathogens was assessed through co-culture experiments. The probiotic strains were co-cultured with *S. mutans*, *F. nucleatum*, *P. gingivalis*, and *P. intermedia* in broth media at 37 °C under anaerobic conditions. The experiment was conducted with modifications based on the method described by Kang et al. [16]. For co-cultivation with *S. mutans* KCTC 3065, a culture medium composed of a 1:1 mixture of de Man, Rogosa, and Sharpe (MRS) broth supplemented with 0.05% L-cysteine and brain heart infusion (BHI) broth was utilised. In the co-culture experiments involving *F. nucleatum*KCTC 2640, *P. gingivalis* KCTC 5352, and *Prevotella intermedia* KCTC 15693, a 1:1 mixture of MRS broth (0.05% L-cysteine) and KCOM1 broth was employed.

The prepared culture medium was inoculated with *Lactobacillus gasseri* HHuMIN D and *L. paracasei* OK in combination, along with the respective oral pathogenic bacteria, to achieve a final concentration of 1 × 10^7^ CFU/mL. The cultures were then incubated under anaerobic conditions at 37 °C for 18–24 hours. To quantify bacterial counts, 1 mL of the culture medium was sampled, and the resulting cell pellet was collected for analysis. The primers used for bacterial quantification are detailed in Table 1. Bacterial counts were quantified using real-time PCR after incubation, and the inhibition rate was calculated by comparing bacterial counts in the treated group with those in the control.

#### Inhibition of pathogen adhesion to oral epithelial cells

To evaluate the inhibitory effect of *Lactobacillus gasseri* HHuMIN D and *L. paracasei* OK on the adhesion of oral pathogenic bacteria to host cells, the experimental procedure was conducted with modifications based on the method described by [12]. Human oral epithelial KB cells (Korean Cell Line Bank 10017) were utilised for the assay. The KB cells were cultured in RPMI 1640 medium (Roswell Park Memorial Institute 1640; Welgene, Daegu, Korea) supplemented with 300 mg/L L-glutamine, 10% foetal bovine serum (FBS), 100 µg/mL streptomycin, and 100 U/mL penicillin under controlled conditions of 5% CO₂ at 37 °C.

For the bacterial preparation, cultures of *L. gasseri* HHuMIN D, *L. paracasei* OK, and the oral pathogenic bacteria were centrifuged at 4,000 rpm for 10 minutes, and the resulting pellets were re-suspended in RPMI 1640 medium to a final concentration of 1.0 × 10^8^ CFU/mL. KB cells were seeded into 24-well plates (SPL Life Sciences Co., Ltd., Pocheon, Korea) at a density of 3.0 × 10^5^ cells/well and incubated under 5% CO₂ at 37 °C until a confluent monolayer was established. Subsequently, the culture supernatant in each well was removed, and 2 mL of a 1:1:1 mixture of the *L. gasseri* HHuMIN D and *L. paracasei* OK suspension with the oral pathogenic bacteria suspension (1.0 × 10^8^ CFU/mL) was added to the monolayer. The plate was then incubated under 5% CO₂ at 37 °C for 1 hour to allow bacterial adhesion.

Following incubation, the supernatant was removed, and the wells were washed twice with phosphate-buffered saline (PBS). The adhered bacteria and KB cells were then harvested by treating the wells with 200 µL of trypsin-EDTA (Welgene, Daegu, Korea) for 5 minutes. The positive control group underwent the same experimental conditions, except that only the oral pathogenic bacteria suspension (1.0 × 10^8^ CFU/mL) was added to the KB cell monolayer without probiotic treatment. The number of bacteria adhered to the KB cells was quantified by extracting genomic DNA from the harvested pellet and performing real-time PCR analysis.

#### Quantification of bacterial populations via genomic DNA extraction and real-time PCR

To establish a genomic DNA standard curve, *Lactobacillus gasseri* HHuMIN D, *L. paracasei* OK, and oral pathogenic bacteria were individually cultured, and bacterial cells were harvested at concentrations ranging from 1 × 10^8^ to 1 × 10^10^ CFU. Genomic DNA was then extracted using the MagMAX™ Microbiome Ultra Nucleic Acid Isolation Kit (Applied Biosystems) according to the manufacturer’s protocol. The extracted genomic DNA was serially diluted in 10-fold increments, and real-time PCR was performed to obtain Ct values for the construction of standard curves based on bacterial CFU counts.

The PCR reaction mixture was prepared under the following conditions: 2 μL of genomic DNA, 10 μL of PowerSYBR Green PCR Master Mix (Applied Biosystems), 400 nM each of forward and reverse primers, and nuclease-free water to a final volume of 20 μL. Real-time PCR was conducted under the following thermal cycling conditions: an initial denaturation at 95 °C for 10 minutes, followed by 50 cycles of 95 °C for 15 seconds and 60 °C for 1 minute. Bacterial counts were determined by converting the Ct values using the standard curve.

### *In vivo* animal model and experimental design

#### Probiotic administration

To evaluate the effects of probiotics on halitosis and periodontal health, 45 male Sprague-Dawley rats (5 weeks old, 106.9-121.4 g) were obtained from a certified animal supplier and housed under controlled conditions (temperature: 22 ± 1 °C, humidity: 55 ± 10%, and a 12-hour light/dark cycle). The rats were acclimated for one week with free access to standard chow and water. Following acclimatisation, the animals were randomly assigned to five groups (*n* = 9 per group) as illustrated in Figure 2(a). The administered probiotic doses of 0.2, 2, and 20 mg/head/day correspond to approximately 10^7^, 10^8^, and 10^9^ CFU/head/day, respectively, based on the viable cell density of the formulation (~5 × 10^7^ CFU/mg).

Probiotics were suspended in sterile water and administered daily via oral gavage for five weeks. Control groups received equivalent volumes of sterile water without probiotics. Ligature-induced periodontitis was initiated during the experimental period by placing sterile 4-0 silk sutures bilaterally around the maxillary second molars under ketamine (50 mg/kg) and xylazine (5 mg/kg) anaesthesia. Ligature placement was performed three weeks after the start of probiotic administration and maintained for two weeks prior to sacrifice, as shown in Figure 2(a).

#### Outcome measure

##### Body weight monitoring

Body weight was recorded weekly using an electronic balance to assess overall health and detect any adverse effects of ligature placement or probiotic administration.

##### Saliva secretion measurement

Non-stimulated saliva secretion was measured on the final day of the experiment. Rats were anaesthetised using ketamine and xylazine, and cotton swabs were placed at the floor of the oral cavity for 30 minutes. The swabs were weighed before and after saliva collection using a precision microbalance (accuracy: 0.01 mg) to determine the volume of saliva produced.

##### Alveolar bone loss assessment

After euthanasia, the maxillae were dissected, and soft tissue was removed by autoclaving. Samples were stained with 1% methylene blue to visualise the cementoenamel junction (CEJ) and alveolar bone crest (ABC). Images were captured using a stereomicroscope, and CEJ-ABC distances were measured at six predetermined sites per molar using ImageJ software.

##### Tongue coating index (TCI)

The Winkel Tongue Coating Index was used to evaluate tongue coating [17] Tongue images were captured under standardised lighting conditions using a high-resolution digital camera. Tongue coating assessment was consistently performed by the same evaluator throughout the study using the modified TCI scoring criteria. The tongue surface was divided into six sections, and each section was scored based on coating density as follows:

- 0 = No coating
- 1 = Light coating
- 2 = Heavy coating

Scores for all sections were summed to obtain a total TCI score (range: 0–12).

#### Cytokine analysis

Blood samples were collected via cardiac puncture under anaesthesia and centrifuged at 3000 rpm for 15 minutes to isolate serum. Tumour necrosis factor-alpha (TNF-α), interleukin-1 beta (IL-1β) and interleukin-6 (IL-6) levels were quantified using commercial enzyme-linked immunosorbent assay (ELISA) kits (R&D Systems) according to the manufacturer’s instructions. Results were expressed in pg/mL.

#### Microbiome composition

There is increasing evidence that periodontal pathogens commonly enriched in gingival and subgingival niches such as *Fusobacterium nucleatum*, *Porphyromonas gingivalis*, and *Prevotella* spp. also contribute to halitosis through VSC production and proteolytic activity [18,19]. Therefore, this study selected gingival tissue for microbiome analysis to capture the periodontal disease-associated microbial shifts within the ligature-induced periodontitis model. This approach was intended to evaluate the broader pathophysiological context linking periodontal inflammation, microbial dysbiosis, and halitosis-related pathways, rather than to directly characterise the tongue dorsum microbiome. DNA was extracted using a commercial kit (Qiagen). The V3-V4 region of the 16S rRNA gene was amplified and sequenced on an Illumina MiSeq platform. Sequencing data were processed using QIIME2 for microbial diversity analysis, including relative abundance comparisons and principal component analysis (PCA) based on Weighted UniFrac distance metrics.

#### Statistical analysis

All statistical analyses, except microbiome beta-diversity and differential abundance analyses, were performed using SPSS (version 26.0) and R Studio. Data are presented as mean ± standard deviation (SD). Prior to statistical analysis, data normality was assessed using the Shapiro–Wilk test. Because the datasets did not significantly deviate from normality (*p* > 0.05), differences among experimental groups were evaluated using one-way analysis of variance (ANOVA) followed by Tukey’s post hoc multiple-comparison test. Statistical significance was defined as *p* < 0.05. Values reported as ‘N.D.’ indicate concentrations below the assay-specific limit of detection (LOD).

Microbial beta-diversity was assessed using weighted UniFrac distance matrices and visualised by principal coordinates analysis (PCoA). Differences in overall microbial community composition among groups were evaluated using permutational multivariate analysis of variance (PERMANOVA) with 999 permutations implemented through the *adonis2* function in the *vegan* package in R (version 4.5.1).

Differential abundance analysis was conducted using Analysis of Compositions of Microbiomes with Bias Correction (ANCOM-BC) in R. Genus-level count data were analysed with experimental group as the primary explanatory variable. To control for multiple testing, *p*-values were adjusted using the Benjamini–Hochberg false discovery rate (FDR) procedure, and taxa with adjusted *p*-values (*q*-values) < 0.05 were considered significantly differentially abundant. Heatmaps were generated solely for visualisation of microbial community composition patterns and were not used to determine statistical significance or differential abundance.

## Results

### *In vitro* study results

#### Effects of probiotics on *mgl* gene expression

The anaerobic oral bacteria *P. gingivalis* and *F. nucleatum* are established to harbour the *mgl* gene, which encodes the enzyme L-methionine-α-deamino-γ-mercaptomethane-lyase (METase) [20]. This pyridoxal 5'-phosphate-dependent enzyme catalysers the degradation of L-methionine, a sulphur-containing amino acid, into a suite of volatile sulfur compounds (VSCs). These gaseous metabolites, including hydrogen sulphide (H_2_S) and methyl mercaptan (CH_3_SH), are primary contributors to the phenomenon of oral malodor, a significant concern in oral health [20,21].

Given the direct role of the *mgl*/METase pathway in generating the particularly malodorous methyl mercaptan (CH_3_SH) and its prevalence in bacteria associated with both halitosis and periodontitis, this enzyme represents a compelling therapeutic target. Interventions aimed at METase inhibition hold the potential for dual benefits: reducing a primary contributor to oral malodor and attenuating a significant virulence factor in key periodontal pathogens [5,10,21].

**Figure 1.**
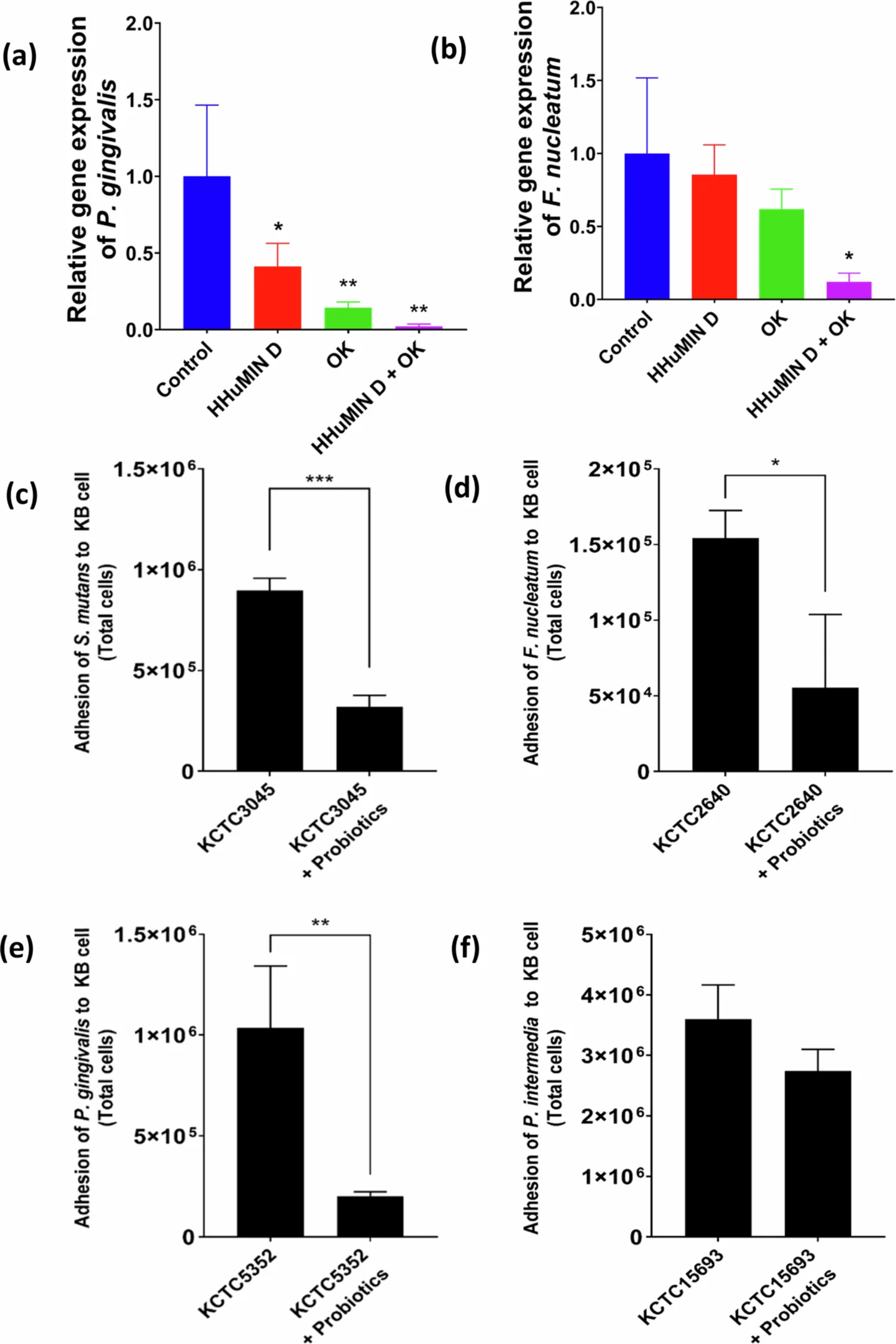
Changes in *mgl* gene expression in *P. gingivalis* (a) and *F. nucleatum* (b) after treatment with culture supernatants of *L. gasseri* HHuMIN D and *L. paracasei* OK, individually and in combination. Data are expressed as mean ± standard deviation from three independent experiments (*p < 0.05, *p < 0.01). Number of adhered pathogenic bacteria in oral epithelial cells following co-culture with *L. gasseri* HHuMIN D and *L. paracasei* OK. (c): *S. mutans*, (b): *F. nucleatum*, (c): *P*. *gingivalis*, (d): *P*. *intermedia*. Values are expressed as the mean ± SEM of three different experiments (*p < 0.05, **p < 0.01, ***p < 0.001).

In this study, *mgl* gene expression in *P. gingivalis* was reduced by 60% and 86% following treatment with *Lactobacillus gasseri* HHuMIN D and *Lactobacillus paracasei* OK culture supernatants, respectively. A 1:1 mixture of these supernatants demonstrated a synergistic inhibitory effect, resulting in a 98% reduction (Figure 1(a)). In *F. nucleatum*, exposure to the culture supernatant of *L. gasseri* HHuMIN D resulted in a modest 15% reduction in *mgl* gene expression. In contrast, *L. paracasei* OK supernatant elicited a more substantial 39% decrease. Notably, a synergistic effect was observed upon treatment with a 1:1 mixture of both supernatants, leading to an 88% reduction in *mgl* expression (Figure 1(b)). The findings demonstrated that a 1:1 combination of *L. gasseri* HHuMIN D and *L. paracasei* OK almost completely inhibited the expression of the *mgl* gene in both *P. gingivalis* and *F. nucleatum*.

#### Effects of probiotics on oral pathogenic bacteria growth and epithelial cell adhesion

The pathogenesis of dental caries and periodontal diseases is orchestrated by the concerted action of specific bacterial species within the dental biofilm [22]. Notably, *Streptococcus mutans*' acidogenic metabolism and exopolysaccharide synthesis initiate caries, its acid tolerance sustaining enamel demineralisation. *F. nucleatum* serves a critical structural role in biofilm maturation through coaggregation, while also contributing to halitosis and an anaerobic environment. As a keystone pathogen in periodontitis, *P. gingivalis* manipulates host immunity and directly degrades periodontal tissues, significantly contributing to volatile sulfur compound (VSC)-related halitosis. The hormone-sensitive proliferation of *Prevotella intermedia* links it to pregnancy gingivitis, with its production of lipopolysaccharide (LPS) and VSCs contributing to inflammation and halitosis. These bacteria exhibit significant synergistic interactions, where *S. mutans* conditions the early biofilm, *F. nucleatum* provides structural support, and *P. gingivalis's* immunomodulatory effects benefit the dysbiotic community, culminating in VSC production and halitosis as a clinical indicator of periodontal inflammation [20,22–24].

Thus, we examined the influence of the probiotic strains *L. gasseri* HHuMIN D and *L. paracasei* OK on the proliferation of the cariogenic and periodontopathic bacteria *S. mutans*, *F. nucleatum*, *P. gingivalis*, and *P. intermedia* and their adhesion to oral epithelial cells. Our findings revealed that the co-administration of these probiotic strains substantially suppressed the growth of these pathogens. Specifically, the probiotic co-culture led to marked reductions in growth relative to the positive control: *S. mutans* (98.2%), *F. nucleatum* (91.2%), *P. gingivalis* (99.1%), and *P. intermedia* (96.6%) (Table 2). Also, the adhesion capabilities of oral pathogenic bacteria to oral epithelial cells were significantly attenuated following treatment with the *L. gasseri* HHuMIN D and *L. paracasei* OK co-culture (Table 3, Figure 1 (c)-(d)). Compared to the control, the percentage reduction in adhesion was as follows: *S. mutans* (64.40%), *F. nucleatum* (64.04%), *P. gingivalis* (80.52%), and *P. intermedia* (23.90%).

**Table 2.** The inhibitory effects of *L. gasseri* HHuMIN D and *L. paracasei* OK co-culture on oral pathogenic bacterial growth.

Strains	Experimental groups	Number of bacteria (cells/mL)	Inhibitory rate (%)
*S. mutans*	*S. mutans* KCTC3065	1.4 × 10^10^ ± 1.1 × 10^9^	
	*S. mutans* KCTC3065 + *L. gasseri* HHuMIN D + *L. paracasei* OK	2.5 × 10^8^ ± 1.2 × 10^8**^	98.2
*F. nucleatum*	*F. nucleatum* KCTC2640	3.5 × 10^4^ ± 2.4 × 10^4^	
	*F. nucleatum* KCTC2640 + *L. gasseri* HHuMIN D + *L. paracasei* OK	3.0 × 10^3^ ± 1.1 × 10^3^	91.2
*P. gingivalis*	*P. gingivalis* KCTC5352	2.4 × 10^9^ ± 3.3 × 10^7^	
	*P. gingivalis* KCTC5352 + *L. gasseri* HHuMIN D*+L. paracasei* OK	2.0 × 10^7^ ± 4.2 × 10^6 ***^	99.1
*P. intermedia*	*P. intermedia* KCTC15693	1.3 × 10^9^ ± 3.9 × 10^7^	
	*P. intermedia* KCTC15693*+ L. gasseri* HHuMIN D*+L. paracasei* OK	4.2 × 10^7^ ± 1.3 × 10^6 ***^	96.6

Values are expressed as the mean ± standard deviation of three different experiments. (**p < 0.01, ***p < 0.001).

**Table 3.** Inhibitory rate of *Lactobacillus gasseri* HHuMIN D and *Lactobacillus paracasei* OK on the adhesion ability of pathogenic bacteria to oral epithelial cells.

Strains	Experimental groups	Number of bacteria (Total cells)	Inhibitory rate (%)
*S. mutans*	*S. mutans* KCTC3045	897246 ± 28537	
	*S. mutans* KCTC3045 + *L. paracasei* OK + *L. gasseri* HHuMIN D	319438 ± 27129^***^	64.40 ± 3.02
*F. nucleatum*	*F. nucleatum* KCTC2640	154196 ± 8665	
	*F. nucleatum* KCTC2640 + *L. paracasei* OK + *L. gasseri* HHuMIN D	55453 ± 22790^*^	64.04 ± 14.78
*P. gingivalis*	*P. gingivalis* KCTC5352	1035773 ± 144433	
	*P. gingivalis* KCTC5352 + *L. paracasei* OK + *L. gasseri* HHuMIN D	201798 ± 9960^**^	80.52 ± 0.96
*P. intermedia*	*P. intermedia* KCTC15693	3601149 ± 265550	
	*P. intermedia* KCTC15693 + *L. paracasei* OK + *L. gasseri* HHuMIN D	2740438 ± 168972	23.90 ± 4.69

### *In vivo* study results

To systematically evaluate the effects of probiotics on halitosis and periodontal health, multiple parameters were assessed, including body weight, saliva secretion, alveolar bone loss, TCI, inflammatory cytokine levels, and microbiome composition.

#### Body weight as an indicator of systemic health

**Figure 2.**
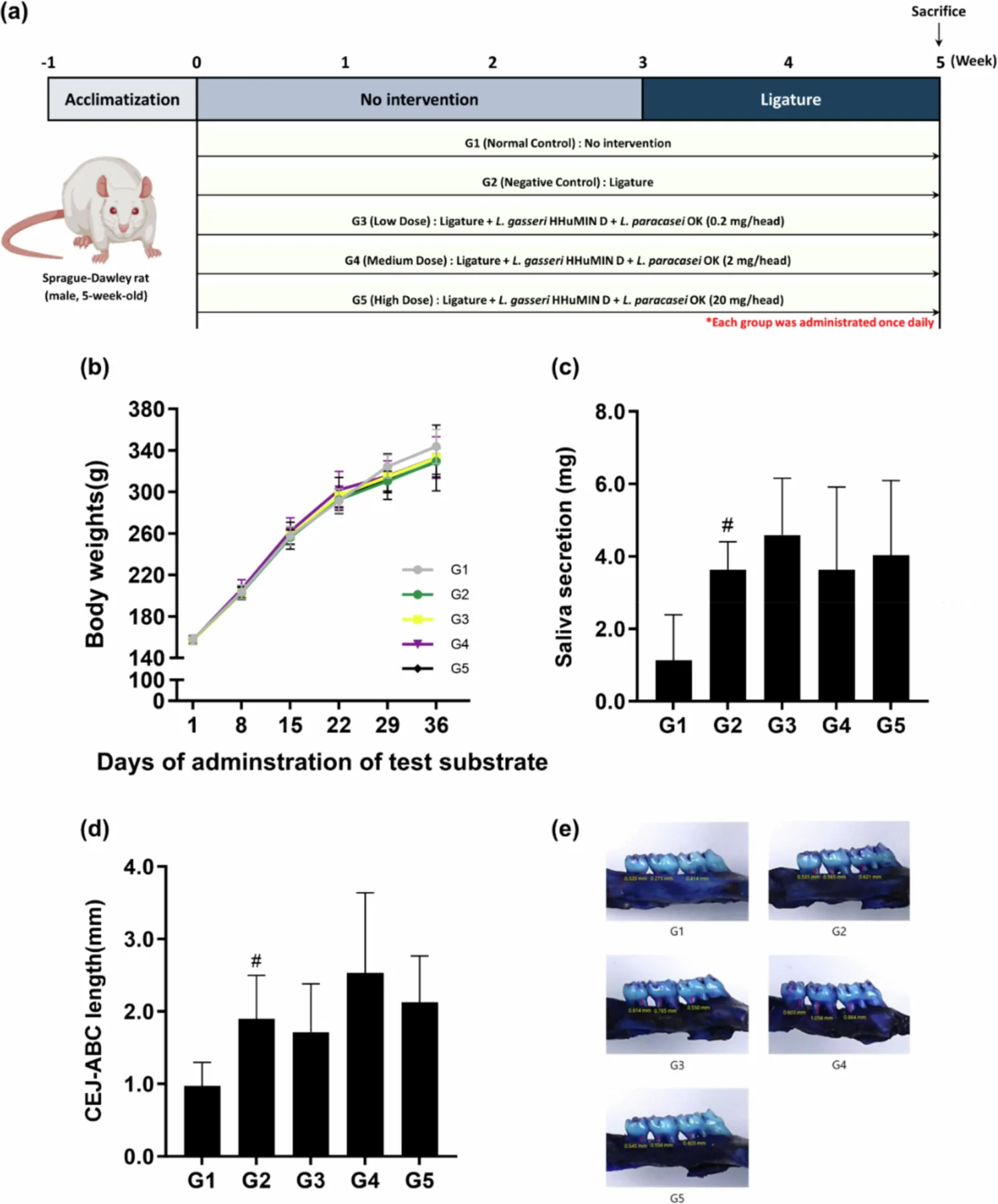
Physiological and histological changes in the ligature-induced periodontitis rat model. (a) The scheme of ligature-induced periodontitis rat model; (b) Body weight changes in response to probiotic administration; (c) Total saliva volume measured to assess salivary secretion; (d) Quantification of alveolar bone loss based on the distance between the cementoenamel junction (CEJ) and alveolar bone crest (ABC); (e) Representative images of methylene blue staining for measuring the CEJ-ABC. G1: Normal Control (No intervention); G2: Negative Control (Ligature-only group to induce periodontitis); G3: Low Dose (Ligature + 0.2 mg/head/day probiotics); G4: Medium Dose (Ligature + 2 mg/head/day probiotics); G5: High Dose (Ligature + 20 mg/head/day probiotics). Significance is indicated as ^#^ *p* < 0.05 compared to G1.

Body weight was monitored as an indicator of overall systemic health and the potential adverse effects of periodontal inflammation. A clear trend was observed across the groups. The negative control group (G2), which underwent ligature-induced periodontitis without probiotic treatment, exhibited a significant weight reduction, with an average final weight of 329.54 ± 12.44 g. In contrast, probiotic-treated groups demonstrated a recovery (Figure 2(b)). This suggests that probiotic supplementation may partially mitigate the systemic impact of periodontal inflammation, possibly by modulating the inflammatory response or improving metabolic health.

#### Saliva secretion as a marker of oral physiological function

Saliva production was evaluated as a key physiological marker of oral health. Non-stimulated saliva secretion was significantly higher in the negative control group (G2) (3.62 ± 0.78 mg), indicating that periodontal inflammation may have impaired salivary gland function, potentially leading to excessive or dysregulated saliva production (Figure 2(c)). No significant changes were observed in the probiotic-treated groups. Non-stimulated saliva secretion differed significantly among experimental groups (Figure 2(c)). The periodontitis control group exhibited significantly higher salivary secretion than the healthy control group (*p* < 0.05). Salivary secretion in the probiotic-treated groups tended to be lower than that of the periodontitis control group and approached the levels observed in the healthy control group; however, no significant differences were detected among the probiotic treatment groups.

#### Alveolar bone loss analysis

Alveolar bone loss, a hallmark of periodontal disease progression, was evaluated by measuring the distance between the cementoenamel junction and alveolar bone crest (CEJ–ABC). The periodontitis control group (G2) exhibited significantly greater alveolar bone loss (1.897 ± 0.601 mm) than the healthy control group (G1) (0.974 ± 0.326 mm) (Figure 2(d)), confirming successful induction of periodontal disease. Although the probiotic-treated groups (G3–G5) showed numerically lower CEJ–ABC distances compared with G2, these differences were not statistically significant (*p* > 0.05). Therefore, under the conditions and duration of the present study, probiotic supplementation did not significantly reduce alveolar bone loss associated with ligature-induced periodontitis.

#### TCI as a direct marker of halitosis

**Figure 3.**
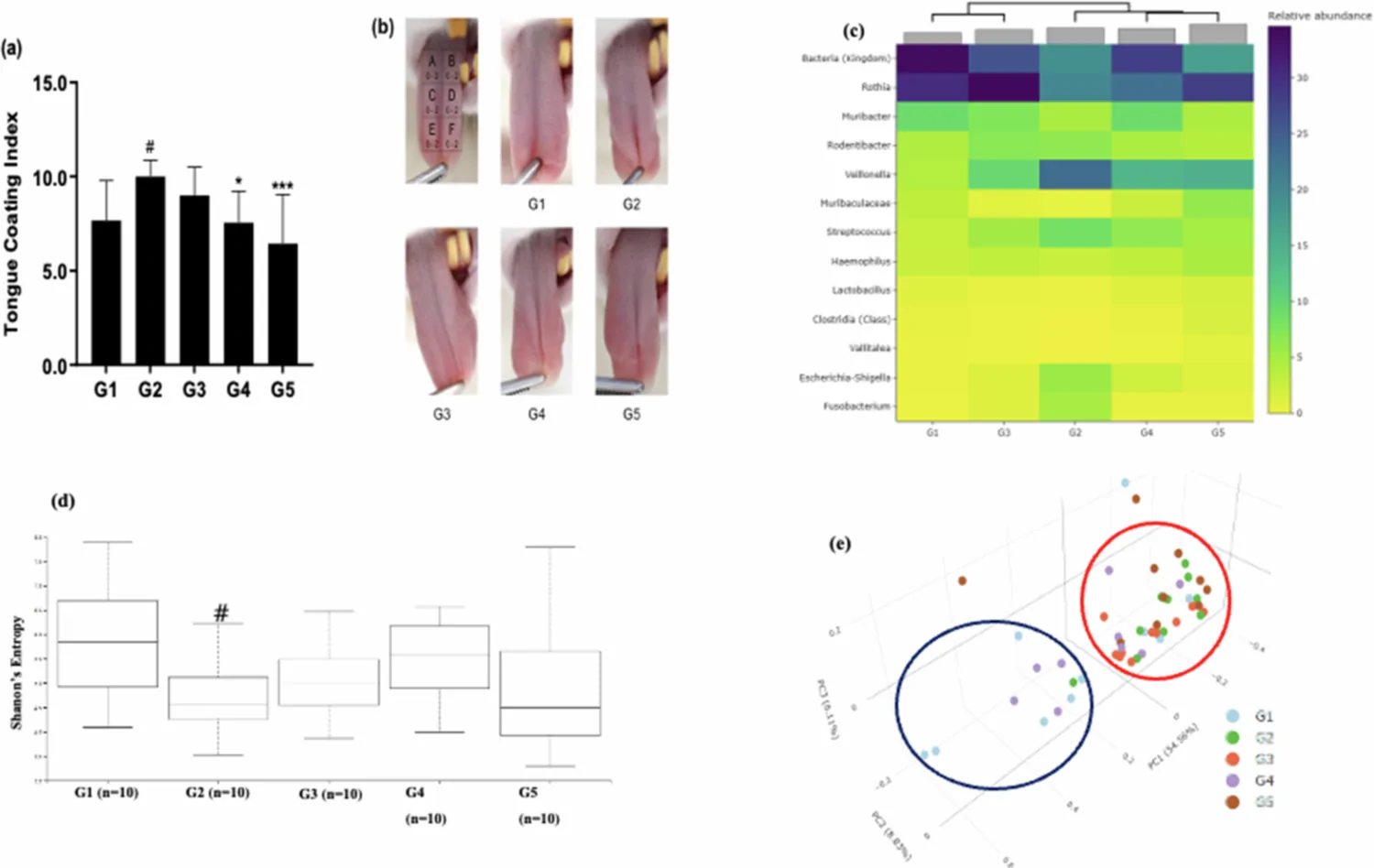
Scoring of Tongue Coating Index in ligature-induced periodontitis rat model. (a) Quantification of tongue coating index by Winkel tongue coating index; (b) Representative tongue coating images. G1: Normal Control (No intervention); G2: Negative Control (Ligature-only group to induce periodontitis); G3: Low Dose (Ligature + 0.2 mg/head/day probiotics); G4: Medium Dose (Ligature + 2 mg/head/day probiotics); G5: High Dose (Ligature + 20 mg/head/day probiotics). Significance is indicated as follows: ^#^ *p* < 0.05 vs. G1, *p < 0.05 vs. G2 and ***p < 0.001 vs. G2. Effect of probiotic administration on oral microbiome composition in ligature-induced periodontitis rat model. (c) Heatmap representation of genus-level bacterial composition across different experimental groups (G1-G5). The color scale indicates relative abundance, with higher values shown in darker colors. (d) Boxplot showing the alpha diversity (Shannon index) of the oral microbiome across different groups. The symbol (#) indicates a statistically significant difference compared to the control group. (e) Principal coordinates analysis (PCoA) based on weighted UniFrac distances illustrating beta diversity among experimental groups. Distinct clustering patterns were observed between ligature-induced periodontitis and normal groups. Statistical differences in microbial community composition were confirmed by PERMANOVA with 999 permutations (pseudo-F = 2.688, R^2^ = 0.193, *p* = 0.003). G1: Normal Control (No intervention); G2: Negative Control (Ligature-only group to induce periodontitis); G3: Low Dose (Ligature + 0.2 mg/head/day probiotics); G4: Medium Dose (Ligature + 2 mg/head/day probiotics); G5: High Dose (Ligature + 20 mg/head/day probiotics). Significance is indicated as follows: ^#^ *p* < 0.05 vs. G1.

The TCI, a well-established clinical marker of halitosis, exhibited notable differences across groups (Figure 3(a)-(b)). The negative control group (G2) demonstrated a significantly elevated TCI score (10.0 ± 0.9), indicating increased bacterial biofilm accumulation associated with periodontal inflammation. In contrast, probiotic supplementation led to a dose-dependent reduction in TCI: Medium-dose probiotic group (G4): 7.6 ± 1.7 and High-dose probiotic group (G5): 6.4 ± 2.6. These results suggest that probiotic supplementation effectively reduces bacterial biofilm formation on the tongue, thereby improving oral hygiene and potentially alleviating halitosis. The observed reduction in TCI may be attributed to the modulation of oral microbiota, particularly the suppression of odour-producing bacterial species.

#### Inflammatory cytokine profile and systemic immune modulation

Inflammatory cytokines were analysed to assess the systemic immunomodulatory effects of probiotics. The low-dose probiotic group (G3) exhibited a significant reduction in IL-1β levels (0.464 ± 1.391 pg/mL) compared to the negative control group (G2) (8.379 ± 5.467 pg/mL) (Table 4). Since IL-1β is a key pro-inflammatory cytokine involved in periodontal disease progression, this reduction suggests that probiotics may help regulate systemic inflammatory responses associated with periodontal infection. However, further investigation is needed to determine whether these effects are sustained over longer periods.

**Table 4.** Inflammatory cytokine concentrations in a ligature-induced periodontitis rat model.

	Experimental groups				
Inflammation biomarkers	Control	Ligature	Ligature + 10^7^	Ligature + 10^8^	Ligature + 10^9^
TNF-α (pg/mL)	0.510 ± 1.468	N.D.	N.D.	N.D.	N.D.
IL-1β	9.003 ± 7.800	8.379 ± 5.467	0.464 ± 1.391*	8.743 ± 5.393	5.131 ± 5.088
IL-6 (pg/mL)	15.970 ± 26.036	9.627 ± 16.184	4.601 ± 9.717	N.D.	17.767 ± 31.855

Values are expressed as the mean ± standard deviation of three different experiments. (*p < 0.05, **p < 0.01, ***p < 0.001); N.D. refers not detected.

Serum cytokine concentrations of ligature-induced periodontitis rat model. Data is expressed as the mean ± standard deviation. One-way analysis of variance (ANOVA) was applied to evaluate differences in discrete variables between the groups.

#### Microbiome composition and pathogenic taxa reduction

To investigate the effects of probiotics on halitosis and the oral microbiome, we analysed the bacterial composition of different experimental groups using a heat-map representation of genus level abundance (Figure 3(c)). In the ligature-induced periodontitis group (G2), there was a notable increase in pathogenic bacteria, including *Fusobacterium, Escherichia-Shigella, Streptococcus*, and *Veillonella*. However, probiotic administration in Groups 3 and 4 (G3-G4) led to a significant reduction in *Fusobacterium* abundance relative to G2, indicating the potential role of probiotics in modulating oral microbiota and mitigating halitosis.

Shannon index analysis was conducted to assess microbial diversity across the groups (Figure 3(d)). The ligature-induced periodontitis group (G2) exhibited significantly lower microbial diversity compared to the control, suggesting a dysbiotic oral environment associated with halitosis. Interestingly, the probiotic-treated groups (G3-G4) maintained microbial diversity levels comparable to the control, indicating that probiotic supplementation may help preserve a balanced oral microbiome.

To further evaluate microbial compositional differences, we performed beta-diversity analysis using weighted clustering. The results revealed distinct clustering patterns between the ligature -induced and control groups, supporting the notion that periodontitis-induced halitosis is associated with significant shifts in microbiome composition (Figure 3(e)). PCoA based on weighted UniFrac distances demonstrated distinct clustering patterns among experimental groups. PERMANOVA analysis confirm ed that microbial community composition differed significantly among groups (pseudo-F = 2.688, R^2^ = 0.193, *p* = 0.004).

Differential abundance analysis was performed using ANCOM-BC to statistically evaluate changes in bacterial taxa among groups. The global ANCOM-BC test indicated that *Fusobacterium* abundance differed significantly among groups (W = 118.30, q-value = 3.55 × 10^−22^). When G2 was used as the reference group, *Fusobacterium* was significantly less abundant in G1, G3, G4, and G5 than in G2. The log fold changes were −5.38 for G1, −1.53 for G3, −2.87 for G4, and −2.97 for G5, with corresponding adjusted *p*-values of 5.82 × 10^−23^, 0.0398, 5.40 × 10^−5^, and 4.71 × 10^−5^, respectively. These results support a statistically significant group-dependent difference in *Fusobacterium* abundance.

## Discussion

### Probiotics suppressed *mgl* gene expression, pathogenic oral bacteria and their adhesion to oral epithelial cells

The probiotic strains *Lactobacillus gasseri* HHuMIN D and *Lactobacillus paracasei* OK demonstrably caused a significant downregulation in the expression of the *mgl* gene. This gene is involved in VSC-associated metabolic pathways. Notably, this reduction in *mgl* gene expression was consistently observed in both *P. gingivalis* and *F. nucleatum*. Furthermore, the combined application of the supernatants from these two probiotic strains exhibited a synergistic inhibitory effect on *mgl* expression within both bacterial species. These findings strongly suggest the therapeutic potential of this probiotic combination in addressing halitosis.

In a prior study, *Weissella cibaria* CMU, a LAB isolated from healthy Korean children’s saliva and commonly found in kimchi, demonstrated a significant 45% reduction in *P. gingivalis mgl* expression compared to the control [10]. Notably, the application of heat-killed *W. cibaria* CMU suppressed methyl mercaptan (CH_3_SH) production through a dual mechanism: inhibition of METase enzymatic activity and downregulation of the *mgl* gene in *P. gingivalis*. In a separate investigation, *Lacticaseibacillus paracasei* ET-22 and its postbiotic constituents, including phenyl lactic acid and the peptide GP[Hyp]GAG, significantly reduced *mgl* expression by 47.7% compared to the control in both *F. nucleatum* and *S. mutans* [21]. In contrast to these studies, the synergistic effect observed in our research with a 1:1 mixture of *L. gasseri* HHuMIN D and *L. paracasei* OK on *mgl* gene downregulation in *P. gingivalis and F. nucleatum* offers a compelling rationale for their application in mitigating halitosis.

In addition, the antimicrobial efficacy of *L. gasseri* HHuMIN D and *Lactobacillus paracasei* OK against a range of oral bacteria, including *S. mutans*, *F. nucleatum*, *P. gingivalis*, and *P. intermedia*, was evident in co-culture assays. These experiments revealed significant inhibitory effects of the *Lactobacillus* strains on the proliferation and their adhesion to oral epithelial cells of each of the tested pathogenic species.

Consistent with these findings, a prior study investigated the antibacterial properties of cell-free *Lactobacillus reuteri* AN417 culture supernatant (LRS) against *P. gingivalis*, *F. nucleatum*, and *S.* mutans [24]. Exposure to LRS resulted in significant reductions in their growth rates, intracellular ATP levels, cell viability, and time-to-kill. The minimal inhibitory volumes (MIVs) of LRS were determined to be 10% (v/v) for *P. gingivalis*, 20% (v/v) for *F. nucleatum*, and 30% (v/v) for *S. mutans*. Although the antibacterial component in the probiotic, tentatively proposed by the authors to be a novel fatty acid or sugar, requires further characterisation, these results underscore the potential of LRS as a bioactive agent against pathogens associated with periodontitis^22^.

In summary, our *in vitro* findings revealed that a 1:1 combination of *L. gasseri* HHuMIN D and *L. paracasei* OK significantly suppressed the expression of the *mgl* gene in *P. gingivalis* and *F. nucleatum*. Furthermore, this probiotic co-culture inhibited the growth and epithelial cell adhesion of pathogenic oral bacteria, indicating the potential of *L. gasseri* HHuMIN D and *L. paracasei* OK as promising candidates for probiotic interventions targeting halitosis.

### Influence of complex OK on halitosis relating to periodontitis, malodor-producing biofilms, and microbial abundance

#### Impact to periodontal inflammation and biofilm formation

The results of this study highlight the multifaceted benefits of Complex OK comprising *Lactobacillus gasseri* HHuMIN D and *Lacticaseibacillus paracasei* OK in managing halitosis and mitigating the effects of periodontitis. The observed improvements in body weight suggest that probiotics may have systemic benefits, potentially counteract the adverse effects of periodontal inflammation and maintain physiological homoeostasis [25].

The increased salivary secretion observed in the periodontitis control group should not be interpreted as evidence of salivary gland dysfunction. Rather, it may reflect an adaptive response to periodontal inflammation and microbial dysbiosis. Oral inflammatory conditions can influence salivary secretion through local inflammatory mediators and neuroimmune signalling pathways [26,27]. In addition, salivary output in animal studies may be affected by factors such as anaesthesia, stress, and sample collection procedures [28,29]. Because only saliva volume was measured in this study, and no assessments of salivary composition, glandular histology, or functional biomarkers were performed, conclusions regarding salivary gland dysfunction cannot be drawn. The tendency of probiotic-treated groups to exhibit salivary secretion levels closer to those of healthy controls may indicate partial normalisation of inflammation-associated alterations in salivary physiology. However, the underlying mechanisms require further investigation.

One of the most compelling findings was the significant reduction in the TCI in the probiotic-treated groups, suggesting that probiotics may reduce tongue coating-associated bacterial biofilm accumulation, a key contributor to halitosis. These results align with previous studies, such as those by Offenbächer et al. [14], which emphasised the role of probiotics in disrupting malodor-producing biofilms and modulating oral microbiota. This study further supports the growing body of research suggesting that probiotics may serve as a novel adjunctive therapy for halitosis management.

However, despite the positive outcomes observed in halitosis-related parameters, no significant improvement in alveolar bone loss was detected among probiotic-treated groups. This finding is likely attributable to the short duration of the study, as bone remodelling and regeneration require a longer time frame and, in many cases, adjunctive therapies such as mechanical debridement or osteoinductive agents. Future research should explore the long-term effects of probiotics on bone regeneration, potentially in combination with other therapeutic approaches.

#### Microbiome modulation and host immune response

Microbiome analysis provided key insights into the potential mechanisms underlying the beneficial effects of probiotics. The high-dose probiotic group exhibited a notable reduction in pathogenic taxa, including *Veillonella*, *Streptococcus*, and *Escherichia-Shigella*. These bacterial species have been associated with halitosis and periodontal disease progression through VSC-associated metabolic activity and pro-inflammatory responses. The observed modulation of microbial communities suggests that probiotics may exert their beneficial effects by competing with pathogenic bacteria, producing antimicrobial peptides, and enhancing host immune responses.

Although probiotics were administered via oral gavage rather than direct topical application to the oral cavity, previous studies have suggested that oral–gut microbiome crosstalk and systemic immunomodulatory pathways may indirectly influence oral microbial ecology and inflammatory responses [30,31]. The oral cavity and gastrointestinal tract are interconnected microbial habitats, and bidirectional oral–gut microbial interactions have been reported to modulate microbial composition and host physiological responses under various inflammatory conditions [30]. In addition, microbiome-targeted interventions have been proposed as potential approaches for regulating chronic inflammatory diseases, including periodontitis, through host–microbiome interactions and immune modulation [31]. Accordingly, the observed improvements in tongue coating indices, reductions in Fusobacterium abundance, and decreased IL-1β levels observed in the present study are consistent with these proposed mechanisms but do not provide direct evidence that oral–gut crosstalk or systemic immune modulation mediated these effects. Because gut microbiota, systemic immune pathways, and probiotic colonisation were not directly assessed, these mechanisms should be regarded as biologically plausible hypotheses rather than experimentally verified pathways. Future studies incorporating gut microbiome profiling, immunological analyses, and mechanistic investigations will be required to establish the causal pathways underlying these observations.

One of the key strengths of this study is its comprehensive approach, integrating physiological, microbiological, and immunological analyses to elucidate the effects of probiotics on halitosis and periodontal health. The dose-dependent responses observed further support the potential for optimising probiotic formulations to enhance their therapeutic efficacy.

However, several limitations must be acknowledged. The relatively short experimental duration may have restricted the observation of long-term effects, particularly in terms of bone regeneration and sustained microbiome shifts. Additionally, while the ligature-induced periodontitis model in rats provides a highly controlled experimental setting, it does not fully replicate the complexities of human oral microbiota and host immune responses. In addition, tongue coating assessment may involve observer-dependent interpretation because the Winkel Tongue Coating Index was originally developed for clinical evaluation in humans rather than animal models. Furthermore, although *mgl* downregulation and improvements in tongue coating indices suggested potential modulation of halitosis associated pathways, direct quantification of volatile sulfur compounds (VSCs) was not performed in this study. Therefore, future studies using analytical approaches such as gas chromatography or halimeter measurements are needed to confirm the direct effects of Complex OK on VSC production. Future studies should also focus on longer experimental durations and the inclusion of diverse probiotic strains to better understand the long-term effects and translational potential of probiotics in human oral health management.

### Dual mechanisms underlying the synergistic action of complex OK

This study demonstrates that Complex OK exerts complementary anti-halitosis effects through both *in vitro* and *in vivo* models. The strongest mechanistic evidence was obtained from the *in vitro* experiments, which directly demonstrated suppression of oral pathogens, inhibition of epithelial cell adhesion, and downregulation of the *mgl* gene involved in VSC-associated metabolic pathways. The *in vivo* findings further supported the biological relevance of these mechanisms by showing improvements in tongue coating, modulation of the oral microbiome, and reduced IL-1β levels in the ligature-induced periodontitis model. Specifically, *in vitro*, Complex OK achieved approximately 98% inhibition of *P. gingivalis* and 88% inhibition of *F. nucleatum*, significantly exceeding the effects of either strain used alone. This enhanced efficacy suggests a genuine synergistic interaction, possibly due to complementary antimicrobial mechanisms. *Lactobacillus* spp. is known to produce diverse inhibitory substances such as organic acids, hydrogen peroxide, and bacteriocins, which may act in concert to suppress oral pathogens [22]. For instance, *Weissella cibaria* secretes hydrogen peroxide, which inhibits *F. nucleatum* growth and thereby reduces the production of VSCs [11].

It is conceivable that in Complex OK, each probiotic contributed distinct antimicrobial metabolites, such as hydrogen peroxide or reuterin, resulting in more potent suppression of odour-associated bacteria than either strain could achieve alone. Supporting this hypothesis, previous studies have demonstrated that cell-free supernatants from *Lactobacillus salivarius* and *L. reuteri* can inhibit the growth and coaggregation of *P. gingivalis* and *F. nucleatum*, reinforcing the hypothesis that probiotic combinations may disrupt both the viability and interaction of oral pathogens [32].

*In vivo*, oral administration of Complex OK was associated with favourable modulation of the oral microbiome, including a reduction in *Fusobacterium* abundance, together with improvements in tongue coating indices. These findings support the biological relevance of the *in vitro* observations, although they should be interpreted as supportive rather than direct mechanistic evidence.

Consistent with the *in vitro* findings discussed above, Complex OK significantly downregulated *mgl* expression in both *P. gingivalis* and *F. nucleatum*, suggesting modulation of VSC-associated metabolic pathways. Previous studies reporting suppression of *mgl* by natural compounds further support the feasibility of targeting this pathway as a strategy for managing halitosis. Notably, prior studies have demonstrated that natural compounds like epigallocatechin gallate (EGCg) can reduce CH₃SH production by inhibiting *mgl* expression in *P. gingivalis* [33]. Our findings extend this paradigm to probiotic interventions, suggesting that beneficial microbes can also downregulate odour-related genes, potentially offering a more selective and microbiota-friendly alternative to traditional antiseptics.

Additionally, data from our team’s human intervention study provides valuable insight into the systemic potential of Complex OK. These findings suggest that Complex OK may alleviate halitosis not solely via local antimicrobial action but also through systemic pathways involving enhanced glucose and phosphorus metabolism. Of particular significance is the complex’s potential to influence VSCs originating in the gastrointestinal tract, which are often overlooked in oral-targeted therapies. This suggests that Complex OK may exert dual-site efficacy in both the oral cavity and gastrointestinal tract, thereby broadening its clinical applicability for systemic halitosis.

**Figure 4.**
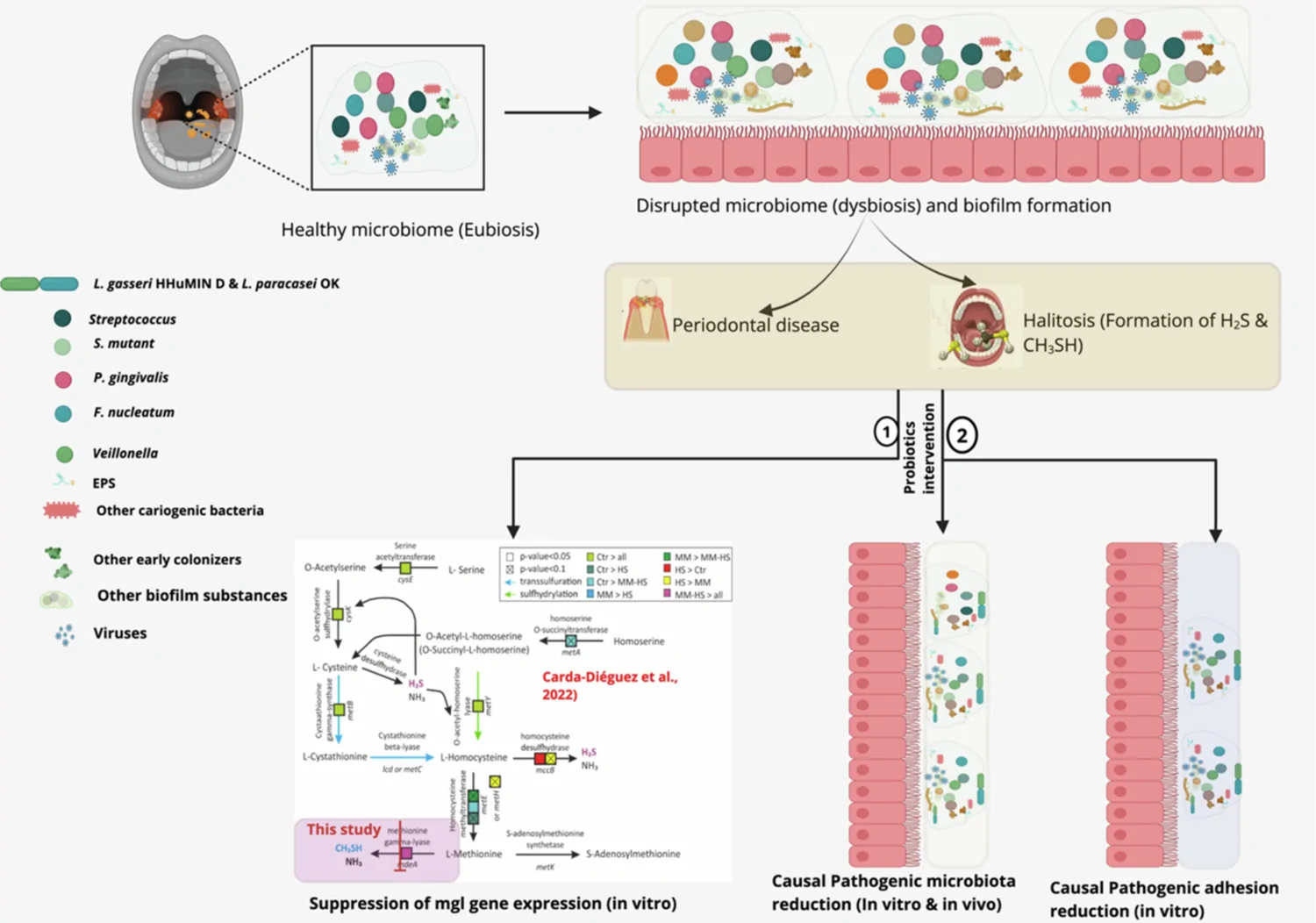
Process of Halitosis formation and the process of remediation by probiotics intervention (Modified from Carda-Dieguez et al. [34].

Taken together, the present findings indicate that Complex OK exerts anti-halitosis effects through complementary microbial and molecular mechanisms. The mechanistic basis of these effects was primarily established through the *in vitro* experiments, whereas the *in vivo* findings provided supportive evidence of biological activity through modulation of oral microbial composition, inflammatory responses, and tongue coating indices. These results support the potential of Complex OK as a promising probiotic candidate for managing halitosis and periodontal health, while additional studies incorporating direct VSC measurements and clinical validation are warranted (Figure 4).

## Limitations

Several limitations should be considered when interpreting the present findings. First, the study did not include a positive control group, such as chlorhexidine or a conventional antimicrobial treatment, which limits direct comparison of Complex OK with established therapeutic approaches. Second, volatile sulfur compounds were not directly quantified *in vivo* using gas chromatography or halimeter-based methods. Therefore, conclusions regarding halitosis-related outcomes are based on indirect evidence, including *mgl* gene downregulation and improvements in tongue coating index, and should be interpreted as modulation of VSC-associated pathways rather than direct evidence of reduced sulfur gas production. Third, microbiome analyses were performed using gingival tissue samples to evaluate periodontal dysbiosis; however, the tongue dorsum is recognised as the principal niche associated with halitosis and VSC generation. Although several taxa identified in gingival tissues are also implicated in oral malodor, gingival microbiota may not fully represent the tongue-associated microbial community. Further studies incorporating positive control treatments, direct VSC measurements, and site-specific tongue dorsum microbiome analyses are warranted to further validate the therapeutic potential of Complex OK for halitosis management.

## Conclusion

This study highlights the promising therapeutic potential of the probiotic combination *L. gasseri* HHuMIN D and *L. paracasei* OK, collectively known as Complex OK, for managing halitosis and enhancing periodontal health. Both *in vitro* and *in vivo* results clearly demonstrated that this probiotic duo significantly suppressed the expression of the *mgl* gene demonstrated that this probiotic combination downregulated *mgl*, a gene associated with volatile sulfur compound (VSC)-related metabolism, in *P. gingivalis* and *F. nucleatum*, two major oral pathogens associated with halitosis and periodontitis. In addition to gene suppression, Complex OK remarkably inhibited the growth and adhesion of various pathogenic oral bacteria, highlighting its potential to interfere with microbial colonisation and biofilm formation two key factors in the persistence of oral malodor and periodontal disease. *In vivo* administration in a rat model not only improved tongue coating indices, reduced systemic IL-1β levels, and shifted the oral microbiome toward a more balanced microbial composition. Although the probiotic treatment did not significantly reverse alveolar bone loss within the study period, the observed immunomodulatory and microbiota-stabilising effects suggest that long-term supplementation could contribute to sustained oral health benefits. Collectively, these findings support the potential application of Complex OK as a microbiome-friendly probiotic approach for managing oral conditions associated with halitosis and periodontal inflammation. Future studies involving longer treatment durations and human clinical trials will be essential to confirm these benefits and further explore its translational potential in oral healthcare.

## Data Availability

The raw 16S rRNA gene sequencing FASTQ files and associated metadata generated during this study have been deposited in the NCBI Sequence Read Archive (SRA) under BioProject accession number **PRJNA1470849**. These data are publicly available through the NCBI SRA repository.
